# Simultaneous external validation of various cardiac arrest prognostic scores: a single-center retrospective study

**DOI:** 10.1186/s13049-021-00935-w

**Published:** 2021-08-14

**Authors:** Takumi Tsuchida, Kota Ono, Kunihiko Maekawa, Takeshi Wada, Kenichi Katabami, Tomonao Yoshida, Mineji Hayakawa

**Affiliations:** 1 Department of Emergency Medicine, Hokkaido University Hospital, N14W5 Kita-ku, Sapporo, 060-8648 Japan; 2 Ono Biostat Consulting, Naritahigashi, Suginami-ku, Tokyo, 166-0015 Japan

**Keywords:** Out-of-hospital cardiac arrest, Prediction, Prognosis, Neurological outcome

## Abstract

**Background:**

This study aimed to compare and validate the out-of-hospital cardiac arrest (OHCA); cardiac arrest hospital prognosis (CAHP); non-shockable rhythm, unwitnessed arrest, long no-flow or long low-flow period, blood pH < 7.2, lactate > 7.0 mmol/L, end-stage chronic kidney disease, age ≥ 85 years, still resuscitation, and extracardiac cause (NULL-PLEASE) clinical; post-cardiac arrest syndrome for therapeutic hypothermia (CAST); and revised CAST (rCAST) scores in OHCA patients treated with recent cardiopulmonary resuscitation strategies.

**Methods:**

We retrospectively collected data on adult OHCA patients admitted to our emergency department between February 2015 and July 2018. OHCA, CAHP, NULL-PLEASE clinical, CAST, and rCAST scores were calculated based on the data collected. The predictive abilities of each score were tested using the area under the curve (AUC) of the receiver operating characteristic (ROC) curve.

**Results:**

We identified 236 OHCA patients from computer-based medical records and analyzed 189 without missing data. In OHCA patients without bystander witnesses, CAHP and OHCA scores were not calculated. Although the predictive abilities of the scores were not significantly different, the NULL-PLEASE score had a large AUC of ROC curve in various OHCA patients. Furthermore, in patients with bystander-witnessed OHCA, the NULL-PLEASE score had large partial AUCs of ROC from sensitivity 0.8–1.0 and specificity 0.8–1.0.

**Conclusions:**

The NULL-PLEASE score had a high, comprehensive predictive ability in various OHCA patients. Furthermore, the NULL-PLEASE score had a high predictive ability for good and poor neurological outcomes in patients with bystander-witnessed OHCA.

## Background

Out-of-hospital cardiac arrest (OHCA) occurs annually in 250,000–300,000 patients worldwide [1]. The management of cardiac arrest, including modern cardiopulmonary resuscitation (CPR), extracorporeal CPR, emergency cardiovascular treatment, and targeted temperature management, is progressing [2]; however, in patients with a successful return of spontaneous circulation (ROSC), in-hospital survival and neurologically intact survival rates remain disappointingly low [3].

Accurate prognostication of survival and good neurological outcome after ROSC is very important to reduce unnecessary treatments and counselling the patients’ families. Several clinical scores have been reported to predict the neurological outcome of patients with OHCA at an early stage [4–9]. In 2006, the OHCA score was the first practical score developed to predict prognosis in OHCA patients at intensive care unit admission and has been used for many years [4]. This score was developed based on OHCA-patient data from 1999 to 2003. Therefore, the OHCA score may not reflect recent changes in resuscitation strategies and improvements in the outcome. The cardiac arrest hospital prognosis (CAHP) score was designed to more accurately recognize neurological prognosis with a nomogram, including an independent prognostic factor, and was presented in 2016 [6]. The OHCA and CAHP scores require “no-flow interval,” which is the time from cardiac arrest to the initiation of CPR, to calculate the prediction scores. Therefore, they cannot be applied to patients without witnesses of cardiac arrest [4, 6]. The “non-shockable rhythm, unwitnessed arrest, long no-flow or long low-flow period, blood pH < 7.2, lactate > 7.0 mmol/L, end-stage chronic kidney disease, age ≥ 85 years, still resuscitation, and extracardiac cause” (NULL-PLEASE) clinical score was devised to identify patients unlikely to survive out-of-hospital cardiac arrest using several unfavorable cardiac arrest- or patient-related characteristics [7]. Recently, the post-cardiac arrest syndrome for therapeutic hypothermia (CAST) score and revised CAST (rCAST) score were developed to predict the neurologic prognosis in patients after resuscitation following cardiac arrest, prior to inducing therapeutic hypothermia [10, 11]. The variables required to calculate each predictive score are summarized in Table 1 [4, 6, 7, 10, 11].

**Table 1:** The variables needed to calculate each predictive score

Predictive scores	Age	Initial rhythm upon arrival of EMS	Time from cardiac arrest to CPR	Time from CPR start to ROSC	pH of arterial blood	Lactate	Motor score on the GCS upon arrival at the hospital	Other
OHCA score		○	○	○		○		Serum creatinine
NULL-PLEASE score	○	○		○	○	○		Witness of cardiac arrest
								Bystander CPR
								CPR upon arrival at the hospital
								Cause of cardiac arrest
								End stage renal failure
CAST score		○		○	○	○	○	Serum albumin
								Hemoglobin
								Attenuation on brain CT
rCAST score		○			○	○	○	Time from witnessed time to ROSC
CAHP score	○	○	○	○	○			Epinephrine dose
								Site where cardiac arrest occurred

All laboratory data (pH, lactate, albumin, hemoglobin, serum creatinine) were taken on arrival at the hospital

*OHCA score* out-of-hospital cardiac arrest score, *NULL-PLEASE score* non-shockable rhythm, unwitnessed arrest, long no-flow or long low-flow period, blood pH < 7.2, lactate > 7.0 mmol/L, end-stage chronic kidney disease, age ≥ 85 years, still resuscitation, and extracardiac cause score

*CAST score* post-cardiac arrest syndrome for therapeutic hypothermia score, *rCAST score* revised post-cardiac arrest syndrome for therapeutic hypothermia score, *CAHP score* cardiac arrest hospital prognosis score, *EMS* emergency medical services, *CPR* cardiopulmonary resuscitation, *ROSC* return of spontaneous circulation, *GCS* Glasgow coma scale, *CT* computed tomography

Although various scores have been proposed, it is unclear which prognostication tool is superior in guiding decision-making regarding individual patients with OHCA. Therefore, this study aimed to compare and validate the OHCA, CAHP, NULL-PLEASE clinical, CAST, and rCAST scores in OHCA patients treated with recent CPR strategies.

## Methods

### Patient selection and data collection

This single-center retrospective study evaluated electronic medical records from Hokkaido University Hospital, a tertiary care center in Sapporo City, Japan, which covers 1121 km^2^ with a population of approximately 2.0 million. The study protocol was approved by our institutional review board, and the requirement for informed consent was waived owing to the retrospective design.

Patients with OHCA who were admitted to our emergency department (ED) between February 2015 and July 2018 were included in the present study. Patients were excluded based on the following criteria: (a) age < 18 years, (b) death at the ED, and (c) transferred from other hospitals. From the electronic medical records, we collected pre-hospital data recorded according to the Utstein style [12–14] and in-hospital data regarding CPR, laboratory tests, and treatments. Furthermore, neurological outcomes were evaluated using the cerebral performance category (CPC) scale [15] at 1 month after cardiac arrest. The primary outcome was defined as a good neurological outcome (CPC 1 and 2).

### Statistical analysis

Data for continuous variables are presented as medians with interquartile ranges. Categorical data are presented as frequencies and percentages. Patient characteristics and outcomes were compared between the two groups using the Mann–Whitney U test (for numerical variables) and Fisher’s exact test (for categorical variables). The overall predictive abilities of the various scores for good neurological outcome were tested using the area under the curve (AUC) of the receiver operating characteristic (ROC) curve. To evaluate the predictive ability for good neurological outcome with high specificity, the partial AUC (pAUC) of the ROC curve from a specificity of 0.8–1.0 in each predictive score was calculated. Furthermore, to evaluate the predictive ability for poor neurological outcome with high sensitivity, the pAUC of the ROC curve from sensitivity 0.8–1.0 in each predictive score was calculated. All analyses were performed using R statistical software version 3.6.3 (The Institute of Statistical Mathematics, Tokyo, Japan). All reported *p* values were two-tailed, and differences with *p* < 0.05 were considered statistically significant.

## Results

During the study period, 236 OHCA patients were admitted to our ED. This study included patients with extrinsic cardiac arrest and thus included all OHCA patients regardless of the cause of cardiac arrest. We excluded 47 patients because the neurological outcome 1 month after cardiac arrest was unclear. Therefore, 189 patients were included in the present study. The characteristics of patients with good (CPC 1 and 2) and poor (CPC 3, 4, and 5) neurological outcomes are presented in Table 2.

**Table 2:** Baseline characteristics of the out-of-hospital cardiac arrest patients

Variables	Good (n = 33)	Poor (n = 156)	*p* value
Male, n (%)	22 (66.7%)	90 (57.7%)	0.214
Age, year	62 (54–69)	72 (55–83)	0.023
Cause of cardiac arrest, n (%)			< 0.001
Intrinsic cause			
Cardiac	29 (87.9%)	48 (30.8%)	
Cerebrovascular	0 (0.0%)	11 (7.1%)	
Other	3 (9.1%)	51 (32.7%)	
External cause			
Asphyxia	1 (3.0%)	27 (17.3%)	
Neck hanging	0 (0.0%)	9 (5.8%)	
Trauma	0 (0.0%)	7 (4.5%)	
Other	0 (0.0%)	3 (1.9%)	
Collapse at public space, n (%)	17 (51.5%)	51 (32.7%)	0.032
Witness by bystander, n (%)	27 (81.8%)	100 (64.1%)	0.046
CPR initiated by bystander, n (%)	27 (81.8%)	77 (49.4%)	< 0.001
Time records of EMS, min			
Time from calling EMS to arrival at the scene	7 (6–8)	7 (6–9)	0.409
Time from arrival at the scene to departure from the scene	14 (9–17)	14 (11–17)	0.403
Time from departure from the scene to arrival at the ED	17 (5–20)	12 (8–17)	0.367
Primary ECG rhythm at the scene, n (%)			< 0.001
VF/pulseless VT	22 (66.7%)	31 (19.9%)	
Pulseless electrical activity	11 (33.3%)	52 (33.3%)	
Asystole	0 (0%)	73 (46.8%)	
No flow time, min	1 (1–1)	1 (1–8)	0.033
Low flow time, min	16 (9–24)	30 (20–45)	< 0.001
Blood gas analysis on arrival at the hospital			
pH	7.225 (7.056–7.335)	6.914 (6.729–7.045)	< 0.001
PaCO_2_ (mmHg)	47.1 (38.6–60.0)	78.4 (53.2–104.5)	< 0.001
PaO_2_ (mmHg)	169.0 (101.0–282.0)	122.0 (59.0–342.5)	0.129
HCO_3_ (mmol/L)	18.0 (15.5–21.4)	15.6 (12.6–19.3)	0.009
Lactate (mmol/L)	7.0 (4.6–12.6)	11.8 (9.4–15.0)	< 0.001
Laboratory data on arrival at the ED			
Hemoglobin (g/dL)	12.8 (11.2–14.6)	11.6 (10.2–13.2)	0.002
Creatinine (mg/dL)	0.9 (0.8–1.0)	1.0 (0.8–1.5)	0.013
Serum albumin (g/dL)	3.7 (3.3–4.0)	3.1 (2.7–3.5)	< 0.001
Comorbid chronic kidney disease, n (%)	1 (3.0%)	15 (9.6%)	0.194
Adrenaline dosage until arrival at the hospital (mg)	0 (0–1)	1 (1–2)	< 0.001
CPR by V-A ECMO, n (%)	7 (21.2%)	17 (10.1%)	0.216
Motor response score of the GCS in the ED after ROSC	1 (1–4)	1 (1–1)	< 0.001
Therapeutic hypothermia, n (%)	17 (51.5%)	83 (53.2%)	0.395

*CPR* cardiopulmonary resuscitation, *EMS* emergency medical services, *ED* emergency department, *ECG* electrocardiogram, *VF* ventricular fibrillation, *VT* ventricular tachycardia, *V-A ECMO* veno-arterial extra corporeal membrane oxygenation, *GCS* Glasgow coma scale, *ROSC* return of spontaneous circulation

In Table 3, the AUCs of the ROC curves of each predictive score in all patients and various subgroups are presented. Because the two predictive scores require no-flow time, which we were only able to evaluate in patients with bystander-witnessed OHCA, AUCs of ROC curves of CAHP and OHCA scores were not evaluated in all patients. In the group of patients in which therapeutic hypothermia was not induced, the predictive ability of the CAST and rCAST scores was low, and the rCAST score showed significantly lower predictive ability than the OHCA, CAHP, and NULL-PLEASE scores (*p* = 0.037, 0.016, and 0.027, respectively). In the other subgroups, all predictive scores had sufficiently large AUCs of ROC curves. Although statistical significance was only observed in the group of patients in which therapeutic hypothermia was induced, the NULL-PLEASE score tended to show a high predictive ability in the overall OHCA patient cohort and the subgroups. Furthermore, when comparing subgroups within the same score, the accuracy of the rCAST score in the therapeutic hypothermia (+) group was significantly higher than that in the overall cohort and therapeutic hypothermia (−) group. (*p* = 0.020 and 0.002, respectively). In subgroup analyses of the other scores, therapeutic hypothermia and veno-arterial extracorporeal membrane oxygenation (VA-ECMO) did not affect the scores’ accuracy.

**Table 3:** Area under the receiver operating characteristic curves of each predictive score in all patients and various subgroups

Predictive scores	Number of patients	Area under the curve (95% confidence interval)
All patients, n = 189		
NULL-PLEASE score	189	0.874 (0.807–0.942)
CAST score	173	0.860 (0.777–0.944)
rCAST score	189	0.770 (0.659–0.880)
Patients with by-stander witnessed out-of-hospital cardiac arrest, n = 127		
NULL-PLEASE score	127	0.873 (0.793–0.952)
CAST score	115	0.805 (0.606–0.914)
rCAST score	127	0.728 (0.604–0.851)*
CAHP score	110	0.829 (0.718–0.940)
OHCA score	125	0.847 (0.773–0.921)
Patients in whom therapeutic hypothermia was induced, n = 100		
NULL-PLEASE score	100	0.893 (0.809–0.978)
CAST score	96	0.913 (0.832–0.994)
rCAST score	100	0.925 (0.860–0.989)
CAHP score	73	0.821 (0.667–0.974)
OHCA score	66	0.865 (0.755–0.976)
Patients in whom therapeutic hypothermia was not induced, n = 89		
NULL-PLEASE score	89	0.853 (0.740–0.965)
CAST score	77	0.797 (0.638–0.955)
rCAST score	89	0.599 (0.407–0.792) **
CAHP score	66	0.868 (0.771–0.965)
OHCA score	74	0.831 (0.736–0.926)
Patients in whom veno-arterial extracorporeal membrane oxygenation was not performed, n = 165		
NULL-PLEASE score	165	0.879 (0.807–0.951)
CAST score	150	0.894 (0.801–0.986)
rCAST score	165	0.753 (0.617–0.889)
CAHP score	122	0.869 (0.776–0.962)
OHCA score	138	0.857 (0.784–0.929)
Patients with age ≤ 65, n = 73		
NULL-PLEASE score	73	0.902 (0.809–0.995)
CAST score	65	0.923 (0.845–1.000)
rCAST score	73	0.948 (0.896–1.000)
CAHP score	49	0.879 (0.763–0.995)
OHCA score	42	0.946 (0.879–1.000)

There was no significant difference between the scores in all subgroups

*p < 0.05 compared with the rCAST score in patients with therapeutic hypothermia

**p < 0.01 compared with the rCAST score in patients with therapeutic hypothermia

In patients with bystander-witnessed OHCA, the pAUC of the ROC curve from sensitivity 0.8–1.0 in each predictive score was presented to evaluate the predictive ability for a poor neurological outcome with high sensitivity (Table 4). Although the pAUC of each predictive score was not statistically different, the pAUCs of NULL-PLEASE and CAHP scores were larger than those of the other scores.

**Table 4:** Predictive ability for poor neurological outcome in patients with by-stander witnessed out-of-hospital cardiac arrest

Predictive scores	Number of patients	Partial area under the curve from sensitivity 0.8–1.0	95% confidence interval
NULL-PLEASE score	127	0.1151	0.0577–0.1627
CAST score	115	0.0775	0.0310–0.1307
rCAST score	127	0.0409	0.0000–0.1048
CAHP score	110	0.0759	0.0279–0.1508
OHCA score	125	0.1198*	0.0850–0.1556

*p < 0.05 compared with the rCAST score

To evaluate the predictive ability for good neurological outcome with high specificity, the pAUCs of ROC curves from specificity 0.8–1.0 in each predictive score are presented in Table 5. The pAUCs of NULL-PLEASE and OHCA scores were large, whereas that of the rCAST was small. Furthermore, the pAUC of the OHCA score was statistically larger than that of the rCAST score (*p* = 0.0204).

**Table 5:** Predictive ability for good neurological outcome in patients with by-stander witnessed out-of-hospital cardiac arrest

Predictive scores	Number of patients	Partial area under the curve from specificity 0.8–1.0	95% confidence interval
NULL-PLEASE score	127	0.1175	0.0813–0.1535
CAST score	115	0.1068	0.0698–0.1456
rCAST score	127	0.0831	0.0493–0.1202
CAHP score	110	0.1163	0.0763–0.1550
OHCA score	125	0.0895	0.0542–0.1303

There was no significant difference between the scores

## Discussion

To date, several scores that predict the prognosis of patients with cardiac arrest have been published; however, no study has used the same patient group to verify the accuracy of each score at one time. The present study was the first to simultaneously validate various cardiac arrest prognostic scores. All prognostic scores that were evaluated in the present study had a sufficiently high predictive ability. Among them, the NULL-PLEASE score could be easily calculated in various OHCA patients, including those without bystander witnesses. Furthermore, the NULL-PLEASE score had a high predictive ability for good and poor neurological outcome in patients with bystander-witnessed OHCA.

Previous studies have revealed various factors, such as older age, cardiac arrest occurring at home, initial rhythm other than ventricular tachycardia/ventricular fibrillation, longer duration of no flow, longer duration of low flow, treatment with adrenaline (epinephrine), pupillary response, and a serum lactate level, as prognostic factors for OHCA patients [16–20]. Despite the prognosis scores proposed using these predictors [4–9], prognostication of OHCA patients remains challenging, and no single risk-assessment tool has been recommended for the prognostic classification of OHCA patients. Although various prognostic scores have been reported, the target patients were different in each instance [4–9]. For example, the targets for the NULL-PLEASE score were all OHCA patients, whereas those for the OHCA and CAHP scores were restricted to bystander-witnessed OHCA patients [4, 6, 7]. The targets of the CAST and rCAST scores were restricted to OHCA patients in whom therapeutic hypothermia was induced [10, 11]. However, where possible, we were able to evaluate the prognostic scores in various OHCA patient subgroups, regardless of the original targets.

The prognostic scores require various variables for their calculation. Although no-flow time is required in OHCA and CAHP scores, the variable cannot be obtained in OHCA patients without bystander witnesses [4, 6]. Therefore, OHCA and CAHP scores could not be calculated in OHCA patients without bystander witnesses [4, 6]. In addition, some scores require information that may be difficult to obtain, such as medical history, neurological findings, and findings of brain computed tomography [5, 7, 9, 10]. For these reasons, score calculation is often complicated and/or impossible. In the present study, the OHCA score was the simplest, whereas the CAST score was the most troublesome.

We examined the accuracy of the aforementioned predictive scores across all OHCA patients and various subgroups. As a result, the NULL-PLEASE score had a high, comprehensive predictive ability in all OHCA patients and various subgroups. Furthermore, it had a high predictive ability for good and poor neurological outcome in patients with bystander-witnessed OHCA. Moreover, the NULL-PLEASE score can apply to various OHCA patients because all the variables required to calculate it can be easily collected in clinical settings. Therefore, the NULL-PLEASE score is a useful predictive score in various clinical settings.

Originally, the CAST and rCAST scores were targeted at OHCA patients in whom therapeutic hypothermia was induced [10, 11]. In clinical settings, OHCA patients who have regained consciousness or are strongly predicted to have a poor prognosis tend to be excluded from therapeutic hypothermia. Therefore, the characteristics of OHCA patients who undergo therapeutic hypothermia tend to be restrictive. Furthermore, in OHCA patients who underwent therapeutic hypothermia, the prior probability for good or poor neurological outcome was completely different from that in all OHCA patients. In the present study, although CAST and rCAST scores had high predictive ability in OHCA patients who underwent therapeutic hypothermia, this ability was not observed in other OHCA patients, especially those who did not undergo therapeutic hypothermia. The pAUC from sensitivity 0.8–1.0, which indicates the predictive ability for poor neurological outcome, of the rCAST score in the therapeutic hypothermia (+) group was also significantly higher than those in the overall patient cohort and the therapeutic hypothermia (−) group (data not shown). Therapeutic hypothermia had no effect on the accuracy of the other scores. These results were likely affected by the differences in the aforementioned prior probability. Therefore, rCAST score should not apply patients who were not induced therapeutic hypothermia.

Although CAST and rCAST scores can be calculated online (http://www.castscore.sakura.ne.jp/), other predictive scores cannot. Furthermore, almost all variables required to calculate predictive scores were the same. Therefore, we created a website to conveniently calculate and compare multiple prognostic scores for OHCA patients (https://hokudai-qq.com/score). Comparing different scores simultaneously and selecting the appropriate score for patients with cardiac arrest will be helpful in clinical settings. However, although these scores are useful in explaining the prognosis to relatives, they are not perfectly accurate and should not be used as the basis for clinical decision-making.

### Limitation

This study was conducted retrospectively in a single institution, and the number of target patients was small. In addition, there was potential for selection bias and confounding due to unknown or unmeasured variables. In addition, about 20% of patients were excluded due to unclear follow-up status, leading to inclusion bias.

## Conclusions

Among the predictive scores evaluated in the present study, the predictive abilities of the scores were not significantly different; nevertheless, that of the NULL-PLEASE score was high in various OHCA patients. Furthermore, the variables used to calculate the NULL-PLEASE score can be easily collected in clinical settings. Therefore, the NULL-PLEASE score is a useful predictive score in clinical settings.

## Data Availability

Data are available from the corresponding author upon reasonable request.

## Abbreviations

AUC: Area under the curve
CAHP: Cardiac arrest hospital prognosis
CAST: Cardiac arrest syndrome for therapeutic
CPC: Cerebral performance category
CPR: Cardiopulmonary resuscitation
ED: Emergency department
NULL-PLEASE: Non-shockable rhythm, unwitnessed arrest, long no-flow or long low-flow period, blood pH < 7.2, lactate > 7.0 mmol/L, end-stage chronic kidney disease, age ≥ 85 years, still resuscitation, and extracardiac cause
OHCA: Out-of-hospital cardiac arrest
pAUC: Partial area under the curve
rCAST: Revised CAST
ROC: Receiver operating characteristic
ROSC: Return of spontaneous circulation
VA-ECMO: Veno-arterial extracorporeal membrane oxygenation
